# A Special Section of Articles From the 7^th^ Annual Bio-Ontologies Meeting “Ontologies and Bio-Images”

**DOI:** 10.1002/cfg.441

**Published:** 2004


					Comparative and Functional Genomics
A Special section of articles from
The 7th
Annual Bio-Ontologies Meeting
``Ontologies and Bio-Images''
A Special Interest Group (SIG) Workshop of the
12th Intelligent Systems for Molecular Biology (ISMB)
&
3rd European Conference on Computational Biology (ECCB)
Held at the Moat House Hotel, Glasgow, 30th July 2004
With editorial assistance from
Robert Stevens and Phillip Lord
Workshop Organisers
Robert Stevens (Department of Computer Science, University of Manchester, UK)
Robin McEntire (GlaxoSmithKline, King of Prussia, USA)
Phillip Lord (Department of Computer Science, University of Manchester, UK)
James A. Butler (GlaxoSmithKline, King of Prussia, USA)
The Bio-Ontologies workshop has been a satellite meeting to the annual ISMB conference
since 1998, and is now operated as a Special Interest Group at the ISMB Conference. Bio-
Ontologies is well established as one of the key meetings for dissemination of latest information
and research on ontologies in the life sciences and regularly draws the key researchers in the
field.

				

